# Tuberculosis — United States, 2019

**DOI:** 10.15585/mmwr.mm6911a3

**Published:** 2020-03-20

**Authors:** Noah G. Schwartz, Sandy F. Price, Robert H. Pratt, Adam J. Langer

**Affiliations:** ^1^Division of Tuberculosis Elimination, National Center for HIV/AIDS, Viral Hepatitis, STD, and TB Prevention, CDC; ^2^Epidemic Intelligence Service, CDC.

Since 1989, the United States has pursued a goal of eliminating tuberculosis (TB) through a strategy of rapidly identifying and treating cases and evaluating exposed contacts to limit secondary cases resulting from recent TB transmission (*1*). This strategy has been highly effective in reducing U.S. TB incidence (*2*), but the pace of decline has significantly slowed in recent years (2.2% average annual decline during 2012–2017 compared with 6.7% during 2007–2012) (*3*). For this report, provisional 2019 data reported to CDC’s National Tuberculosis Surveillance System were analyzed to determine TB incidence overall and for selected subpopulations and these results were compared with those from previous years. During 2019, a total of 8,920 new cases were provisionally reported in the United States, representing a 1.1% decrease from 2018.* TB incidence decreased to 2.7 cases per 100,000 persons, a 1.6% decrease from 2018. Non–U.S.-born persons had a TB rate 15.5 times greater than the rate among U.S.-born persons. The U.S. TB case count and rate are the lowest ever reported, but the pace of decline remains slow. In recent years, approximately 80% of U.S. TB cases have been attributed to reactivation of latent TB infection (LTBI) acquired years in the past, often outside the United States (*2*). An expanded TB elimination strategy for this new decade should leverage existing health care resources, including primary care providers, to identify and treat persons with LTBI, without diverting public health resources from the continued need to limit TB transmission within the United States. Partnerships with health care providers, including private providers, are essential for this strategy’s success.

Health departments in the 50 U.S. states and the District of Columbia (DC) report all TB cases that meet the Council of State and Territorial Epidemiologists’ surveillance case definition^†^ to CDC. Reports include patient demographics, clinical features, and medical and social risk factors. Self-reported race/ethnicity data are collected and reported following federal standards; Hispanics/Latinos can be of any race, and all other reported race categories are non-Hispanic/Latino. The U.S. Census Bureau defines a U.S.-born person as one born in the United States or a U.S. territory or born abroad to a U.S. citizen parent. Rates (cases per 100,000 persons) were calculated for the United States and administrative divisions (i.e., the 50 states, DC, and census divisions) using midyear U.S. Census Bureau population estimates.^§^ Rates by national origin and race/ethnicity were calculated using midyear Current Population Survey estimates.^¶^ Average annual percentage changes (APC) in incidence were calculated for 2007–2012 and 2012–2019; these years were selected based on previous research demonstrating a statistically significant change in incidence trends during 2007 and 2012 (*3*). Data regarding drug-resistant TB cases are reported for 2018, the most recent year for which complete drug-resistance data are available.

U.S. TB incidence decreased an average of 2.1% per year during 2012–2019, a slower rate of decline than the average 6.4% per year during 2007–2012. The overall U.S. TB rate for 2019 was 2.7 cases per 100,000 persons, while state-specific 2019 TB rates ranged from 0.2 (Wyoming) to 8.1 (Alaska) (Table 1). Nine states (Alaska, California, Georgia, Hawaii, Maryland, New Jersey, New York, Texas, and Washington) and DC reported TB rates higher than the national rate. Four states (California, Florida, New York, and Texas) continued to account for approximately half of all reported TB cases.

**TABLE 1:** Tuberculosis (TB) case counts and rates with annual percentage changes, by U.S. Census division and state or district — United States, 2018 and 2019

Census division/State	No. of reported TB cases*			TB rate^†^		
	2018	2019	% change	2018	2019	% change^§^
**Division 1: New England**						
Connecticut	51	67	31.4	1.4	1.9	31.6
Maine	14	19	35.7	1.0	1.4	35.2
Massachusetts	200	179	–10.5	2.9	2.6	–10.6
New Hampshire	12	6	–50.0	0.9	0.4	–50.2
Rhode Island	20	14	–30.0	1.9	1.3	–30.1
Vermont	5	3	–40.0	0.8	0.5	–40.0
**Subtotal**	**302**	**288**	**–4.6**	**2.0**	**1.9**	**–4.7**
**Division 2: Middle Atlantic**						
New Jersey	291	311	6.9	3.3	3.5	6.9
New York	744	754	1.3	3.8	3.9	1.7
Pennsylvania	213	198	–7.0	1.7	1.5	–7.1
**Subtotal**	**1,248**	**1,263**	**1.2**	**3.0**	**3.1**	**1.4**
**Division 3: East North Central**						
Illinois	319	327	2.5	2.5	2.6	2.9
Indiana	116	108	–6.9	1.7	1.6	–7.4
Michigan	108	132	22.2	1.1	1.3	22.2
Ohio	178	150	–15.7	1.5	1.3	–15.8
Wisconsin	49	51	4.1	0.8	0.9	3.8
**Subtotal**	**770**	**768**	**–0.3**	**1.6**	**1.6**	**–0.3**
**Division 4: West North Central**						
Iowa	49	52	6.1	1.6	1.6	5.9
Kansas	28	38	35.7	1.0	1.3	35.6
Minnesota	172	147	–14.5	3.1	2.6	–15.0
Missouri	80	70	–12.5	1.3	1.1	–12.7
Nebraska	27	17	–37.0	1.4	0.9	–37.3
North Dakota	13	18	38.5	1.7	2.4	37.7
South Dakota	12	16	33.3	1.4	1.8	32.4
**Subtotal**	**381**	**358**	**–6.0**	**1.8**	**1.7**	**–6.4**
**Division 5: South Atlantic**						
Delaware	22	19	–13.6	2.3	2.0	–14.4
District of Columbia	36	24	–33.3	5.1	3.4	–33.7
Florida	591	558	–5.6	2.8	2.6	–6.6
Georgia	271	301	11.1	2.6	2.8	10.0
Maryland	210	212	1.0	3.5	3.5	0.8
North Carolina	196	185	–5.6	1.9	1.8	–6.6
South Carolina	86	80	–7.0	1.7	1.6	–8.1
Virginia	205	190	–7.3	2.4	2.2	–7.7
West Virginia	6	10	66.7	0.3	0.6	67.8
**Subtotal**	**1,623**	**1,579**	**–2.7**	**2.5**	**2.4**	**–3.5**
**Division 6: East South Central**						
Alabama	91	87	–4.4	1.9	1.8	–4.7
Kentucky	65	66	1.5	1.5	1.5	1.4
Mississippi	81	58	–28.4	2.7	1.9	–28.3
Tennessee	139	128	–7.9	2.1	1.9	–8.7
**Subtotal**	**376**	**339**	**–9.8**	**2.0**	**1.8**	**–10.2**
**Division 7: West South Central**						
Arkansas	76	63	–17.1	2.5	2.1	–17.3
Louisiana	105	89	–15.2	2.3	1.9	–15.0
Oklahoma	74	72	–2.7	1.9	1.8	–3.1
Texas	1,124	1,153	2.6	3.9	4.0	1.3
**Subtotal**	**1,379**	**1,377**	**–0.1**	**3.4**	**3.4**	**–1.1**
**Division 8: Mountain**						
Arizona	178	184	3.4	2.5	2.5	1.7
Colorado	64	66	3.1	1.1	1.1	1.9
Idaho	15	7	–53.3	0.9	0.4	–54.3
Montana	5	2	–60.0	0.5	0.2	–60.3
Nevada	69	52	–24.6	2.3	1.7	–25.9
New Mexico	41	40	–2.4	2.0	1.9	–2.6
Utah	18	27	50.0	0.6	0.8	47.5
Wyoming	1	1	0.0	0.2	0.2	–0.2
**Subtotal**	**391**	**379**	**–3.1**	**1.6**	**1.5**	**–4.4**
**Division 9: Pacific**						
Alaska	63	59	–6.3	8.6	8.1	–5.9
California	2,097	2,118	1.0	5.3	5.4	0.9
Hawaii	120	99	–17.5	8.4	7.0	–17.2
Oregon	81	70	–13.6	1.9	1.7	–14.3
Washington	190	223	17.4	2.5	2.9	16.0
**Subtotal**	**2,551**	**2,569**	**0.7**	**4.8**	**4.8**	**0.4**
**Total**	**9,021**	**8,920**	**–1.1**	**2.8**	**2.7**	**–1.6**

* Based on data from the National Tuberculosis Surveillance System as of March 3, 2020.

^†^ Cases per 100,000 persons. Calculated using midyear population estimates from the U.S. Census Bureau.

^§^ Calculated using unrounded figures.

Among 8,920 TB cases reported during 2019, a total of 6,322 (70.9%) occurred among non–U.S.-born persons (Table 2). From 2018 to 2019, the rate among U.S.-born persons declined 4.2% (to 0.9 cases per 100,000 persons), while the rate among non–U.S.-born persons declined 1.5% (to 14.1) (Table 2) (Figure).

**TABLE 2:** Tuberculosis (TB) case counts and rates, by national origin and race/ethnicity — United States, 2016–2019

U.S. population group	No. of cases* (rate^†^)			
	2016	2017	2018	2019
**U.S.-born^§^ persons**				
Hispanic/Latino	593 (1.6)	582 (1.5)	589 (1.5)	628 (1.6)
White	904 (0.5)	790 (0.4)	807 (0.4)	756 (0.4)
Black/African American	1,057 (3.0)	999 (2.8)	950 (2.7)	905 (2.5)
Asian	144 (2.1)	134 (1.9)	137 (1.9)	120 (1.6)
American Indian/Alaska Native	110 (5.1)	91 (3.8)	102 (4.0)	79 (3.4)
Native Hawaiian/Pacific Islander	30 (4.1)	45 (6.5)	42 (5.6)	23 (3.5)
Multiple or unknown race/ethnicity	22 (—^¶^)	28 (—^¶^)	31 (—^¶^)	42 (—^¶^)
**Subtotal**	**2,860 (1.0)**	**2,669 (1.0)**	**2,658 (1.0)**	**2,553 (0.9)**
**Non–U.S.-born persons**				
Hispanic/Latino	1,976 (10.0)	1,959 (9.9)	2,039 (10.3)	2,065 (10.2)
White	281 (3.7)	266 (3.4)	261 (3.2)	250 (3.1)
Black/African American	911 (22.7)	899 (22.2)	846 (20.3)	825 (19.5)
Asian	3,055 (27.2)	3,128 (27.3)	3,069 (26.0)	3,000 (25.7)
American Indian/Alaska Native	1 (2.9)	2 (2.9)	2 (3.5)	3 (5.3)
Native Hawaiian/Pacific Islander	46 (12.7)	67 (22.7)	72 (24.4)	81 (25.1)
Multiple or unknown race/ethnicity	64 (—^¶^)	52 (—^¶^)	70 (—^¶^)	98 (—^¶^)
**Subtotal**	**6,334 (14.7)**	**6,373 (14.7)**	**6,359 (14.3)**	**6,322 (14.1)**
Unknown national origin	5 (—^¶^)	7 (—^¶^)	4 (—^¶^)	45 (—^¶^)
**Total**	**9,199 (2.8)**	**9,049 (2.8)**	**9,021 (2.8)**	**8,920 (2.7)**

* Based on data from the National Tuberculosis Surveillance System as of March 3, 2020.

^†^ Cases per 100,000 persons. Rates according to national origin and race/ethnicity were calculated using midyear population estimates from the Current Population Survey. Total rate was calculated using midyear population estimates from the U.S. Census Bureau.

^§^ U.S.-born persons were those born in the United States or U.S. territories (American Samoa, Northern Mariana Islands, Guam, Puerto Rico, or U.S. Virgin Islands) or born elsewhere to a U.S. citizen. Non–U.S.-born persons were born outside the United States and U.S. territories, and include those born in the sovereign freely associated states (Federated States of Micronesia, Marshall Islands, or Palau) unless one or both parents were U.S. citizens.

^¶^ Rates could not be calculated for these categories because population estimates are not available.

**FIGURE:**
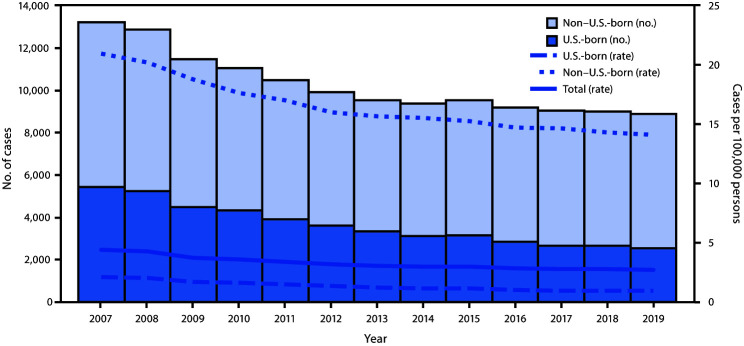
Tuberculosis (TB) case counts and rates, by national origin*^^,†^^ — United States, 2007–2019 * Number of cases with unknown national origin not shown (range = 2–60 per year; median = 7). Total rate includes cases with unknown national origin. ^^†^^ Rates for non–U.S.-born and U.S.-born persons were calculated using Current Population Survey estimates. Total rate was calculated using U.S. Census Bureau population estimates.

Among non–U.S.-born persons residing in the United States, TB rates during 2019 were highest among Asians (25.7 per 100,000), followed by Native Hawaiians/Pacific Islanders (25.1), blacks/African Americans (19.5), Hispanics/Latinos (10.2), and American Indians/Alaska Natives (5.3) and were lowest among whites (3.1) (Table 2). Rates decreased from 2018 to 2019 for all non–U.S.-born groups except American Indians/Alaska Natives and Native Hawaiians/Pacific Islanders. The top five countries of birth among non–U.S.-born persons with incident TB in 2019 were Mexico (1,165 cases; 18.4% of non–U.S.-born cases), the Philippines (790; 12.5%), India (573; 9.1%), Vietnam (503; 8.0%), and China (387; 6.1%).

Among U.S.-born persons, 2019 rates were highest for Native Hawaiians/Pacific Islanders (3.5), followed by American Indians/Alaska Natives (3.4), blacks/African Americans (2.5), Hispanics/Latinos (1.6), and Asians (1.6) and were lowest among whites (0.4). TB incidence decreased from 2018 to 2019 for all U.S.-born groups except Hispanics.

Human immunodeficiency virus (HIV) status was known for 87.3% of reported 2019 TB cases; 4.9% of those patients were coinfected with HIV, including 7.8% of persons aged 25–44 years. Initial drug-susceptibility testing results for at least isoniazid and rifampin were reported for 94.9% of culture-confirmed cases during 2018, the most recent year for which complete data are available.** Among the 6,746 cases during 2018 with available drug-susceptibility test data, 102 (1.5%) were multidrug-resistant^††^; 88 (86.3%) of these cases were among non–U.S.-born persons; 83 (81.4%) reported no previous TB episode. One case of extensively drug-resistant TB^§§^ was reported during 2018; this case occurred in a non–U.S.-born person with a reported previous episode of TB disease.

## Discussion

Since adoption of the U.S. TB elimination strategy in 1989 (*1*), TB incidence has decreased by approximately two thirds (*2*), demonstrating the effectiveness of efforts during the last three decades to prevent TB transmission in the United States. However, the pace of progress has slowed since 2012 (*3*). This slowing is primarily related to the declining proportion of TB cases caused by recent transmission within the United States, against which the U.S. TB elimination strategy has been most effective (*4*). Currently, approximately 80% of TB cases result from reactivation of LTBI acquired years in the past, often outside the United States (*2*).

This shift in U.S. TB epidemiology from being driven primarily by recent transmission within the United States to reactivation of LTBI acquired in the past (often outside the United States) requires an expanded strategy that increases emphasis on detecting and treating LTBI. However, this expanded focus on LTBI cannot compromise existing efforts to prevent TB transmission if the United States is to avoid another TB resurgence, as occurred in the late 1980s and early 1990s (*5*). The U.S. Preventive Services Task Force and CDC recommend routine LTBI screening for populations at increased risk, including persons who have lived in countries with increased TB prevalence and persons who have resided in high-risk congregate settings (e.g., homeless shelters or correctional facilities) (*6*). The efficacy and cost-effectiveness of LTBI screening and treatment, when implemented in populations at risk, compare favorably with other widely accepted preventive care interventions, including mammography to screen for breast cancer (*7*) and use of statins to prevent cardiovascular disease (*8*). LTBI screening (and treatment as indicated) should therefore be considered a routine and integral part of primary care for patients at elevated risk for LTBI.

The findings in this report are subject to at least four limitations. First, this analysis is based on provisional case counts for 2019; however, in previous years, final case counts and rates have not differed greatly from the provisional figures. Second, rates were calculated using estimated population denominators; as a result, rates might change slightly as population estimates are refined in the future. Third, incidence trends for some demographic groups with few patients, e.g., non–U.S.-born American Indian/Alaska Natives, should be interpreted cautiously because of the increased volatility in these rates. Finally, complete drug susceptibility test data are not available for 2019 because susceptibility testing might take several weeks to complete because of the slow-growing nature of *Mycobacterium tuberculosis*.

Concerns regarding the potential adverse effects of LTBI treatment have been an important barrier to LTBI screening and treatment in the past (*9*). To address these concerns, CDC and the National Tuberculosis Controllers Association have released new guidelines that recommend short-course, rifamycin-based regimens, which have less toxicity and better completion rates than does isoniazid monotherapy (*10*). CDC will continue to support and encourage public health partners and primary care providers to increase adoption of LTBI testing and treatment guidelines to accelerate progress toward TB elimination.

**Summary**

**What is already known about this topic?**

Tuberculosis (TB) incidence in the United States has steadily declined since 1993, but the pace of decline has slowed in recent years.

**What is added by this report?**

The U.S. TB rate during 2019 declined to 2.7 cases per 100,000 persons, the lowest level on record. However, the annual pace of decline (−1.6% from 2018) remains slow, particularly among TB cases that are attributed to reactivation of latent TB infection (LTBI).

**What are the implications for public health practice?**

To eliminate TB, the United States needs to expand testing and treatment for LTBI while continuing to prevent TB transmission. Partnerships with health care providers, including private providers, are essential for this strategy’s success.

## Footnotes

* This report is limited to National Tuberculosis Surveillance System case reports verified as of March 3, 2020. Updated data will be available in CDC’s annual TB surveillance report later this year.

† https://www.cdc.gov/tb/programs/rvct/instructionmanual.pdf.

§ https://www.census.gov/data/tables/time-series/demo/popest/2010s-national-total.html.

¶ https://www.census.gov/programs-surveys/cps/data/data-tools.html.

** Because initial drug-susceptibility test results for isoniazid and rifampin were only available for 86.4% of culture-confirmed cases during 2019, more complete data from 2018 are presented. Culture-confirmed cases are defined as cases that were culture-positive on a specimen collected ≤2 weeks after starting TB treatment.

†† A case of TB caused by a strain of *Mycobacterium tuberculosis* that is resistant to at least isoniazid and rifampin.

§§ A case of TB caused by a strain of *Mycobacterium tuberculosis* that is resistant to isoniazid, rifampin, any fluoroquinolone, and at least one injectable second-line drug (i.e., amikacin, kanamycin, or capreomycin).
